# Editorial: Irradiation technologies for vaccine development

**DOI:** 10.3389/fimmu.2022.1075335

**Published:** 2023-01-09

**Authors:** Viskam Wijewardana, Sebastian Ulbert, Giovanni Cattoli

**Affiliations:** ^1^ Animal Production and Health Laboratory, Joint Food and Agriculture Organization (FAO)/International Atomic Energy Agency (IAEA) Centre of Nuclear Techniques in Food and Agriculture, International Atomic Energy Agency, Vienna, Austria; ^2^ Department of Vaccines and Infection Models, Fraunhofer Institute for Cell Therapy and Immunology, Leipzig, Germany

**Keywords:** gamma irradiation, e-beam irradiation, livestock, vaccine, zoonotic diseases, immune responses

Vaccine development is of high priority in the control of infectious diseases. The impact of vaccination on health is immense; except for the improvement of drinking water quality, no other approach has had such a major effect on mortality reduction and population growth (Rodrigues and Plotkin). However, despite an increase in our knowledge on host-pathogen interactions and the advancement in various cutting-edge technologies in vaccine design, there is still a dearth of effective vaccines against many human and animal diseases. The need to design and generate vaccines in a shorter period against emerging and re-emerging pathogens that are difficult to control by other means is critical for human and animal welfare. The efforts to control current SARS-CoV-2 pandemic is a prime example. Inactivated whole virus vaccines were the first vaccines developed and applied against SARS-CoV-2 and they are still widely used (around 50% of total vaccines delivered), indicating the value of this traditional method of vaccine development (1). At present, chemical inactivation is the most common method to kill pathogens for vaccine preparation. However, in the last decade the use of irradiation (gamma-, X-ray-, electron beam-irradiation) has been considered as a potential, valid alternative for vaccine development. Inactivation by irradiation has some potentially important advantages over chemical inactivation. The compilation of this Research Topic will attract attention to the state-of-the art of irradiation technology in vaccine development.

The two mini reviews that appear in this collection give a comprehensive overview of the technology including historical developments. Despite the fact that irradiation technology is still primarily in the research and development phase, there is an increasing interest in this area as illustrated in the review paper by Bhatia and Pillai that provides a representative list of 24 patents that have been filed for the creation of irradiated vaccines for human and animal bacterial, viral, and protozoan diseases. In the second review paper, Unger et al. discuss the development of irradiated vaccines against livestock diseases with special reference to initiatives from the Animal Production and Health section (APH) of the Joint FAO/IAEA Centre of Nuclear Techniques in Food and Agriculture at the International Atomic Energy Agency. In this paper, information on the dose of irradiation used in various vaccine preparations is also provided. Both these articles show the science behind the ionizing radiation and its effect on microorganisms. Depending on the dose applied, ionizing radiation can irreparably damage the microbes. Interestingly, the radiation dose can be calibrated in a way that exposed microorganisms are unable to replicate (i.e. to cause infection) but still retain their metabolic activity, resulting in so-called “metabolically active, non-replicating” microorganisms (Hieke and Pillai). The residual functional proteins that yield metabolic activity in these microorganisms induce a broader immune response in the host when vaccinated. This approach was adopted to develop a metabolically active, non-replicating sporozoite vaccine (PfSPZ vaccine) to prevent *Plasmodium falciparum* malaria which is now being tested at phase 2 clinical trials globally (2). While traditionally gamma irradiation was used for the inactivation of pathogens when developing irradiated vaccines, recent technological advances have paved the way for the use of electron (e)-beam or other irradiation techniques in an effort to move away from the use of radioactive material. Moreover, by employing new radio-protectant compounds such as manganese ions (Mn^2+^) and trehalose, immunogenic epitopes have been better preserved during irradiation inactivation (3).

The antigenic variation of the HA1 domain and its resulting antigenic drift has led to reformulating seasonal influenza vaccines with new strains every year (4). This urge for the development of a universal influenza vaccine that must provide long-lasting cross-protective immunity that can induce both B and T cell responses. In veterinary medicine, the availability of safe and effective avian influenza vaccines suitable for mass applications (e.g. aerosol, drinking water) would facilitate the prevention and control of this disease in poultry. Within this collection there are three articles which aim to investigate if an irradiated influenza vaccine could perform better than chemically inactivated vaccines and/or induce a broader protection. Bortolami et al. investigated the protective efficacy of a H9N2 avian influenza vaccine prototype. In this experiment, birds were vaccinated with an irradiated or a chemical inactivated formulation. The irradiated vaccine group performed as well as the chemical inactivated vaccine group upon challenge when the vaccines were given parenterally. Interestingly, when the vaccine was applied by the mucosal route, the irradiated preparation provided 100% protection at a challenge dose of 3 logs while the chemically inactivated one did not. This indicates the possibility of developing mucosal immunity through irradiated inactivated vaccines which is very difficult to achieve with traditional chemical inactivation. Motamedi Sedeh et al. give further support to this aspect. In their H9N2 avian influenza experiments, both methods of inactivation and routes of administration provided similar levels of protection, but cell-mediated immune responses were more pronounce for the irradiated vaccine formulated with trehalose and given through the mucosal route. In a third article by Singleton et al., the conditions for irradiation were investigated in an H1N1 influenza vaccine experiment. They show not only protection from the vaccine strain but also cross-protection through a gamma irradiated vaccine. Interestingly the induction of higher neutralizing antibodies and more effective cytotoxic T cell responses were correlated with higher temperatures during the irradiation process.

In classical antigen presentation, the exogenous antigen is degraded *via* the endosome pathway and is loaded onto major histocompatibility complex (MHC) class II molecules that presents antigens to CD4 T helper (Th) lymphocytes while inefficient in presenting antigens through MHC class I which is needed to activate CD8 (T cytotoxic) lymphocytes (5). Therefore, traditional inactivated vaccines most often do not yield a pronounced cell-mediated immunity. However, irradiation-inactivated Salmonella gallinarum provided an immune response skewed towards Th1 type (higher IgG2b and IgG3 levels) compared to a formalin inactivated vaccine which led to a protection level similar to a live attenuated vaccine when the challenge was done in chicken (Ji et al.). In another irradiated bacterial vaccine experiment in chicken, Dessalegn et al. showed intranasal or intraocular delivery of gamma irradiated *Pasteurella multocida* provided 100% protection reinforced with higher levels of secretory IgA. Inactivated pathogens are easily cleared by cilia and mucus in the intranasal mucosa unlike live attenuated organisms which can replicate. However, if the structure is maintained along with membrane integrity, this will aid adherence and allow the antigen presenting cells to take up the vaccine antigens. Several groups here investigated the structural integrity following sterilizing irradiation of pathogens when used as vaccine candidates. Electron micrography data showed a high degree of structural integrity when optimum irradiation conditions were used to inactivate Influenza virus (Bortolami et al.) African Swine Fever virus (ASFV) (Pikalo et al.) or *Salmonella gallinarum* (Ji et al.). However, in the case of ASFV, although the irradiated vaccine elicited antibodies when delivered intra-muscularly, there was no protection induced. Since it was shown above that several irradiated vaccines do provide better protection when administered through mucosal rather than parenteral routes, the authors could investigate alternative delivery of the vaccine in future experiments.

One of the bottlenecks which holds back the scaling up and commercial production of irradiated vaccines is the safety and containment requirements for gamma irradiators sourced by radio isotopes. More and more groups are now investigating the use of e-beam or x-ray technologies to produce irradiated vaccines (6). Low-energy electron irradiation (LEEI) which consists of electrons accelerated with up to 500 kilo electron volts (keV) very rapidly delivers high doses necessary for pathogen inactivation, but only requires minimal shielding. Finkensieper et al. showed vaccination with three doses of LEEI inactivated tick-borne encephalitis virus provided complete protection from infection and induced higher antibody titers and avidities as compared to the formalin inactivated virus. E-beam technology can also help in producing biological therapeutics. E-beam irradiated inactivated human rotavirus (HRV) was used as antigen in chickens to produce antibodies against HRV. These egg yolk antibodies and serum derived IgY were effective at neutralizing HRV *in-vitro* (Skrobarczyk et al.). In LEEI processes, determination of the absorbed electron dose is challenging due to the limited, material-dependent penetration depth of the accelerated electrons into the matter. As a solution for this, Schopf et al. proposed the use of bacterial suspensions as biological indicators for electron beam doses.

The use of gamma irradiators has been seen as a hindrance in scaling up of irradiated vaccines against viruses and bacteria, which require relatively high doses. In contrast, eukaryotic cells are several orders of magnitudes more susceptible to ionizing radiation, and consequently a biopharmaceutical production process for attenuation by gamma irradiation has been developed (James et al.) to produce PfSPZ vaccine against malaria. This process was used for several hundred irradiation events to produce the PfSPZ vaccine candidate in the last 13 years which generated multiple lots released for pre-clinical studies and clinical trials. By studying another unicellular parasite, Kangethe et al. performed experiments to identify genes that are involved in disease establishment by gamma irradiation of *Trypanosoma evansi.* By subjecting parasites to a lower-dose of irradiation than that needed to stop replication, the genes responsible for the repair of the radiation-induced damage and thus potential virulent factors were identified. The authors propose a strategy for a candidate vaccine by deleting some of these virulent genes in the parasite. Radiation-induced mutations were also applied to attenuate the virulence of Salmonella spp. to develop live vaccine strains (Ji et al.). The selected mutant strain (ATOMSal-L6) was almost 10,000 times less virulent than its parent strain. Moreover, attenuation was maintained for over 10 passages. ATOMSal-L6 induced protective immunity upon intramuscular vaccination of mice. Finally, Porfiri et al. explored the immunomodulatory landscape of replication deficient metabolically active Lactobacilli produced through gamma irradiation to be used as novel vaccine adjuvants. There is increasing understanding of the role of vaccine adjuvants and how the formulation of modern vaccines can be better tailored towards the desired clinical benefits. Thus discovery of novel adjuvants that could activate specific immune pathways will aid in the quest for developing vaccines against challenging pathogens (7).

In summary, this Research Topic highlights some of the latest developments, innovations and understanding of the use of irradiation technologies for vaccine development. It also raised scientific and technical questions that need to be answered in future research including the underlying mechanisms involved in the remaining metabolic activity in lethally irradiated microbial cells, generation of better cell mediated immunity compared to chemical inactivation, use of LEEI in bulk vaccine preparations, the best route to deliver irradiated vaccines, discovery of novel radio-protectant compounds to preserve vaccine antigenicity and stabilization of irradiated vaccine formulations. Research and technical innovation are also needed to transfer irradiation technologies for vaccine development from an applied research sector to production and marketing.

## Author contributions

All authors listed have made a substantial, direct, and intellectual contribution to the work and approved it for publication.
